# The Dynamics of Avian Influenza: Individual-Based Model with Intervention Strategies in Traditional Trade Networks in Phitsanulok Province, Thailand

**DOI:** 10.1155/2016/6832573

**Published:** 2016-03-23

**Authors:** Chaiwat Wilasang, Anuwat Wiratsudakul, Sudarat Chadsuthi

**Affiliations:** ^1^Department of Physics, Faculty of Science, Naresuan University, Phitsanulok 65000, Thailand; ^2^Department of Clinical Sciences and Public Health and the Monitoring and Surveillance Center for Zoonotic Diseases in Wildlife and Exotic Animals, Faculty of Veterinary Science, Mahidol University, Nakhon Pathom 73170, Thailand

## Abstract

Avian influenza virus subtype H5N1 is endemic to Southeast Asia. In Thailand, avian influenza viruses continue to cause large poultry stock losses. The spread of the disease has a serious impact on poultry production especially among rural households with backyard chickens. The movements and activities of chicken traders result in the spread of the disease through traditional trade networks. In this study, we investigate the dynamics of avian influenza in the traditional trade network in Phitsanulok Province, Thailand. We also propose an individual-based model with intervention strategies to control the spread of the disease. We found that the dynamics of the disease mainly depend on the transmission probability and the virus inactivation period. This study also illustrates the appropriate virus disinfection period and the target for intervention strategies on traditional trade network. The results suggest that good hygiene and cleanliness among household traders and trader of trader areas and ensuring that any equipment used is clean can lead to a decrease in transmission and final epidemic size. These results may be useful to epidemiologists, researchers, and relevant authorities in understanding the spread of avian influenza through traditional trade networks.

## 1. Introduction

Avian influenza, commonly called “bird flu,” is an infectious disease that particularly affects birds, caused by the influenza type A virus [1]. However, there are some subtypes such as A (H5N1) and A (H7N9) that have become infectious to humans. Avian influenza can infect humans and other mammalian species. The first known outbreak of avian influenza A (H5N1) virus in humans occurred in Hong Kong in May 1997 [2]. In epidemiology, poultry would be referred to as the reservoir host, from where the disease can spread to other animals [3, 4]. The transmission of the disease in animals is mainly caused by contact with infected feces or direct contact with infected poultry, as may occur when entering an enclosure where such birds are kept. In general, outbreaks of avian influenza usually occur in winter season as this is a suitable climate for the growth of the virus [3]. The avian influenza virus with high public health and economic impacts that infects poultry is a very virulent virus which is caused by the highly pathogenic avian influenza (HPAI) strain in which the mortality rate may be as high as 90%–100% [3, 5]. From epidemiological and molecular evidences, it is suggested that poultry are the source of the H5N1 outbreak in humans [2]. Undoubtedly, the HPAI virus is an emerging virus that has been causing global concern as the mutations of these viruses always occur unpredictably. For example, a new atypical influenza virus subtype H7N9 emerged in Eastern China in 2013 causing 137 confirmed cases with 45 related deaths [6]. This virus is still active and has spread southwards to as far as Malaysia and to northern areas of China [7].

In Thailand, the first outbreak of avian influenza was officially reported in 2004 in Suphanburi Province [8, 9]. The outbreak occurred in both humans and animals, resulting in a major public health concern and seriously affecting the consumption of poultry in the country, impacting prices, markets, and traders, and leading to lower poultry meat exports, depressed domestic prices, decreased production, and reduced profits [10–12]. Since the 2004 outbreak of HPAI H5N1, more than 63 million poultry have been destroyed to halt the spread of the virus [13]. More than 5.3 billion Thai Baht (US$132.5 million) was spent for direct compensation to affected farmers between 2004 and 2005 [12]. Further outbreaks of HPAI occurred in the central region, the lower northern region, and the eastern region [10, 12] and were mainly detected in backyard chickens, and some outbreaks were detected in other types of domestic poultry [12]. Between 2004 and 2006, four major epidemic waves of HPAI H5N1 in poultry occurred followed by some sporadic cases in 2007 and 2008 [14]. There were 25 laboratory-confirmed HPAI H5N1 human cases with 17 deaths throughout the course of epidemics. Unsurprisingly, most of the cases had previous contact with infected poultry especially backyard chickens [15].

To better understand the dynamics of infectious diseases, mathematical modeling has been extensively used over an extended period of time. Epidemiological and biological data may be entered into the mathematical model and the result may be used to predict possible patterns and the dynamics of the spread of the disease [16–18]. In recent years, the network epidemiological approach has become an important tool in analyzing epidemiological data to develop control and intervention measures [19, 20]. This approach is accepted in medical epidemiology, mathematical biology, and research into predicting the potential spread of diseases and epidemics [21, 22]. More recently, it has become valuable to network science, and, in this context, in showing the relationship between disease and the organisms most likely to be involved. The area of traditional trade networks in the infectious spread of avian influenza is receiving considerable attention from researchers in Thailand. Poolkhet et al. analyzed the movements and trading patterns of backyard chickens by using social network analysis with the egocentric approach [9]. Value chain analysis of backyard poultry has been used to study strategies for preventing the spread of the avian influenza virus [23]. The flow of backyard chickens was previously used to analyze traditional trade networks [24]. However, the dynamics of avian influenza on this kind of network in Thailand are poorly understood. Using an individual-based model may be useful in analyzing the contact network and transmission rates of avian influenza and designing targeted interventions for specific individuals within such networks [25]. The interaction between individuals may be analyzed and considered in an individual-based model. This individual-based model approach was used to develop interventions for HPAI H5N1 epidemics in Vietnam in 2013 [26]. This model indicated that live poultry markets play a major role in the HPAI H5N1 dynamics in Hong Kong and Vietnam [26, 27]. The study of potential transmission mechanisms of the HPAI H5N1 virus in domestic cats led to the development of effective strategies to prevent virus transmission to cats and humans. This study succeeded in evaluating risk factors for human infection by HPAI H5N1 [26].

Approximately 86% of HPAI H5N1 infected flocks involved backyard chickens. Most reported outbreaks of the disease in Thailand therefore involved backyard poultry [14, 24]. As the disease is so strongly associated with backyard poultry production, the consequences of the outbreaks in Thailand are considerable [12, 14]. Most Thai farmers and families keep chickens and ducks as a source of food. Traditional festivals often involve the killing and eating of poultry. These birds may also be sold to supplement household income. Phitsanulok Province, recorded the highest number of confirmed HPAI H5N1 cases in chickens in the second wave of HPAI H5N1 epidemic in Thailand [24]. This province is located in the lower northern region of the country. The backyard chicken population was estimated at 2,450,229 birds by the Department of Livestock Development in 2015 [28]. As well as having three supermarket chains, Phitsanulok Province is supplied with vegetables and meat from five permanent traditional markets [23]. HPAI H5N1 outbreaks would therefore result in serious socioeconomic consequences for the poultry industry, social community, farmers' livelihoods, and public health overall.

In this study, the researchers investigate the dynamics of avian influenza on the traditional trade networks in Phitsanulok Province, Thailand, by using the results of social network analysis [24]. An individual-based model with intervention strategies was proposed [26] to assess the patterns of disease spread. We also simulated the dynamics and behavior of HPAI H5N1 to investigate the influence of transmission probability, virus inactivation, and virus disinfection on the network, to find the most effective intervention strategies for the prevention and control of avian influenza.

## 2. Method

### 2.1. Data Sources and Traditional Trade Network

The focus of this study was on the dynamics of avian influenza on traditional trade networks in Phitsanulok Province in the northern region of Thailand. The spread of avian influenza virus was investigated in the traditional trade network applying the methods used by Wiratsudakul et al. [24]. The field data on trading behaviors was collected at the time of interviews (during June to August 2012) to construct a dynamical model of this trading system and to further visualize with a social network. In Thailand, the smallest administrative unit is the village (called* Moo-ban* in Thai). Phitsanulok Province has 1,045 villages and has several poultry farming systems, with backyard chickens being a significant part of the agricultural lifestyle of the region. Backyard chickens are considered as a homogenous population as these chickens are raised without any fences and freely roam around the village. The trade in live backyard chickens is based on the activities of traders who buy chickens from villages to supply traditional fresh markets with chicken meat.

In this network, nodes represent villages (V), villages with household traders (HT), and villages with trader of trader (TT). Links represent the contact between pairs of nodes. The village nodes represent backyard chickens which are raised and sold to traders [24, 29]. Household trader nodes comprise two types: (1) trader-slaughterhouse, where chickens are collected from villages and slaughtered, and (2) household traders who collect chickens from villages and sell them live to trader of trader. The trader of trader nodes involve contact between buyers and traders, where chickens are bought by traders from other traders (household traders), slaughtered, and sold to vendors, restaurants, and other consumers [24]. The nodes in the networks are often called individuals. Each individual may compose one or more links to other individuals, thus referring to the activities between two individuals who may interact between V-HT, TT-HT, and HT-HT.

**Figure 1:**
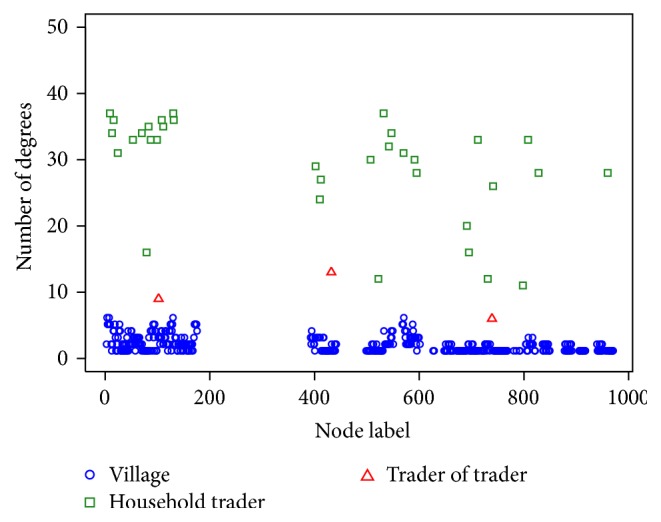
The histogram of degree centrality for 3 kinds of node, that is, villages, household traders, and trader of traders.

All 1,045 villages located in Phitsanulok Province were designated as node labels 1 to 1,045 nodes. However, it was found in [24] that only 467 villages located in an urban area of the province were really connected by the chicken trader network whereas the villages in a remote area organized their trades locally without any connections with the urban network. The node labels 200 to 400, which were located in a remote area, were excluded from the study as the gap in Figure 1. Thus, the traditional trade network in this study consisted of 467 nodes and 953 links [24]: 430 of the nodes were villages with backyard chickens, 34 nodes were villages with household traders and the last 3 nodes were villages with trader of traders. The number of links in each node was also called the degree of the node (Figure 1).

The traditional trade network being analyzed here is termed a scale-free network [30–32] and can be plotted using the power-law degree distribution *P*(*k*) ~ *Ak* ^−*γ*^. It was found in this study that *P*(*k*) ~ 0.7*k* ^−2.1^, where *P*(*k*) is the fraction of nodes having *k* links to other nodes and degree exponent *γ* within the range 2 < *γ* < 3. Such networks exhibit a power-law connectivity distribution. Most real world networks, such as social networks or internet networks, are also considered as scale-free networks. The scale-free connectivity properties are characterized by hubs that may lead to the quick spread of disease in networks where a large degree of the node exists [32]. In this case, most of the elements have very few links, usually characterized by links with and through household traders. These household traders may be very active, therefore making a fairly large number of visits to the village (V) and the trader of trader (TT).

### 2.2. Model

#### 2.2.1. Individual-Based Model

Individual-based modeling was performed on the traditional trade network. In this network, one trader of trader could connect with one or more household traders, while most villages could also connect with one or more household traders (Figure 1). The virus could not transfer directly from villages to trader of traders or vice versa as the trader of trader does not connect directly to a village. Villages cannot connect with each other or with trader of traders without household traders; therefore, the household traders form the links and are the important intermediary in the transmission of disease. The more active the household traders, the greater the number of visits between the villages and the trader of trader, thereby increasing the likelihood that disease may be spread through this link. It is apparent that poultry trade activities play a major role in avian influenza outbreaks and the spread of disease. To be of a more realistic contact structure, a Susceptible-Infectious-Susceptible Model (SIS Model) is incorporated in the individual-based model. For this modeling, the population was divided into two disjoint classes, susceptible individuals (*S*) and infected individuals (*I*). Each node can be either a susceptible or an infected individual. The status of nodes V_*i*,*t*_, HT_*i*,*t*_, or TT_*i*,*t*_ at a given time *t* and node *i* is equal to 1 if contaminated and 0 if not

(1) $$\begin{array}{c} V_{i,t},HT_{i,t},TT_{i,t}=\left\{\begin{array}{cc} 1 & Contaminated\\ 0 & Not\;\;contaminited. \end{array}\right. \end{array}$$

According to the stochastic principle, which holds that the contamination is a random rather than a precise probability, the village and trader of trader could become contaminated if these nodes are visited by at least one contaminated household trader. We define *P*_V,HT_ as the probability that susceptible node V_*i*,*t*_ at time *t* becomes contaminated as a result of the contact with the contaminated nodes HT_*j*,*t*_ when visiting node V_*i*,*t*_ via a link between nodes

(2) $$\begin{array}{c} P_{V,HT}=1-{\left(\;1-P_{HT}\;\right)}^{\sum HT_{j,t}}, \end{array}$$

where *P*_HT_ is the transmission probability of a contaminated household trader transferring the virus when visiting villages and ∑HT_*j*,*t*_ is the summed total of the status of nodes HT_*j*,*t*_ when visiting node V_*i*,*t*_ at time *t*.

*P*_TT,HT_ is the probability that susceptible node TT_*i*,*t*_ becomes contaminated as a result of contact with the contaminated nodes HT_*j*,*t*_ when visiting node TT_*i*,*t*_ via link between nodes

(3) $$\begin{array}{c} P_{TT,HT}=1-{\left(\;1-P_{HT}\;\right)}^{\sum HT_{j,t}}, \end{array}$$

where *P*_HT_ is the transmission probability of a contaminated household trader transferring the virus when visiting a trader of trader and ∑HT_*j*,*t*_ is the summed total of the status of nodes HT_*j*,*t*_ when visiting node TT_*i*,*t*_ at time *t*.

HT_*i*,*t*_ can become a contaminated household trader if they visited a contaminated village or a contaminated trader of trader or they came into contact with a trader of trader at the same time when visiting a contaminated village with probability *P*_HT,V/HT_ and *P*_HT,TT/HT_

(4) PHT,V/HT=1−1−PHT∑Vj,t1−PHH∑HTj,t,PHT,TT/HT=1−1−PHT∑TTj,t1−PHH∑HTj,t,

where *P*_HH_ is the transmission probability of a contaminated household trader transferring virus to another household trader, and ∑V_*j*,*t*_ and ∑TT_*j*,*t*_ are the summed total of the status of nodes V_*j*,*t*_ and nodes TT_*j*,*t*_ visited by node HT_*i*,*t*_ at time *t*, respectively.

A simulation was run on the traditional trade network until the epidemic size reached a steady state, which was achieved after 250 days. Each day was divided into three time steps according to the visit of the householder trader to villages and/or trader of traders. The first infected nodes were located at villages or trader of trader. About 1% of the total villages and 1% of trader of traders were randomly chosen. The choices made were based on the principle that susceptible individuals become infected after contact with infected individuals. Infected individuals will return to the susceptible class after an infective period that was defined as virus inactivation (*T*_*v*_). After transmitting the virus, each infected node (village, trader of trader, or household trader) remained contaminated until the virus became inactivated (*T*_*v*_). A thousand simulations were run for each combination of values of *T*_*v*_ (4–8 days) and of *P*_HT_ and *P*_HH_ (0.3, 0.5, and 0.7). The period of virus inactivation was reported as being between 4 and 8 days [33].

#### 2.2.2. Sequence of Trader of Traders and Villages Visit

The transmission process between the infected individual and the susceptible individual contacts takes place according to the sequence of trader of trader and village visits by household traders. In each day and each simulation, the visit sequence was defined. In this study, only household traders visit villages or trader of traders based on the link of contact, but household traders cannot visit villages or trader of traders simultaneously. Daily, household traders pick up the chickens from the villages and sell them to the trader of traders. A day was divided into 3 time steps according to village and trader of trader visits [24]. The villages and trader of traders, which are visited at each time step, are defined according to stochastic procedures. The sequence matrix is shown as an example matrix. The first three columns are a sequence of visit in each householder trader (the last column), which have 34 householder traders. Thus, the household traders have the chance to meet other household traders elsewhere in the same village or trader of trader in the same time:

(5) $$\begin{array}{c} SM=\left[\begin{array}{cccc} V_{2} & V_{3} & TT_{1} & HT_{1}\\ V_{11} & & TT_{1} & HT_{2}\\ V_{2} & V_{4} & & HT_{3}\\ TT_{2} & V_{2} & & HT_{4}\\ \vdots & \vdots & \vdots & \vdots \\ V_{360} & TT_{3} & V_{362} & HT_{34} \end{array}\right]. \end{array}$$

#### 2.2.3. Virus Disinfection

A purpose of this study was to investigate virus disinfection of an outbreak of avian influenza in traditional trade networks. Virus disinfection is one of the best intervention strategies to prevent and control an outbreak. The means used to control outbreaks differ according to where it occurs. In farms, infected birds may be killed and the corpses are burnt. Infected equipment may be cleaned using a disinfectant spray. Villages, household traders, or trader of traders that are considered as infected can be disinfected each day for the duration of the infection. The period of infection is defined as virus disinfection, *T*_*c*_ (Day). In the modeling activity in this study, the virus disinfection process was performed according to this individual-based model. The optimal virus disinfection period for the prevention and control of an avian influenza outbreak was the main focus of our study. To reduce the spread of the disease through the traditional trade network, we hypothesized that the virus disinfection process could be applied simultaneously to both household traders and trader of traders as the hubs in this network.

#### 2.2.4. Sensitivity Analysis

A sensitivity analysis of the individual-based model was performed by investigating the influence of uncertainty on the changed system. It was necessary to identify the parameters and initial conditions to which the system is the most and least sensitive. Sensitivity analyses were conducted for each transmission probability, *P*_HT_ and *P*_HH_, virus inactivation *T*_*v*_, and virus disinfection *T*_*c*_. To prevent avian influenza from spreading, an intervention process was modeled wherein HTs, TTs, Vs, or both HTs and TTs were disinfected for *T*_*c*_∈ (0 d, 3 d, 4 d, 5 d, 6 d, 7 d, and 8 d).

## 3. Results

### 3.1. Avian Influenza Spread without Intervention Strategies

**Figure 2:**
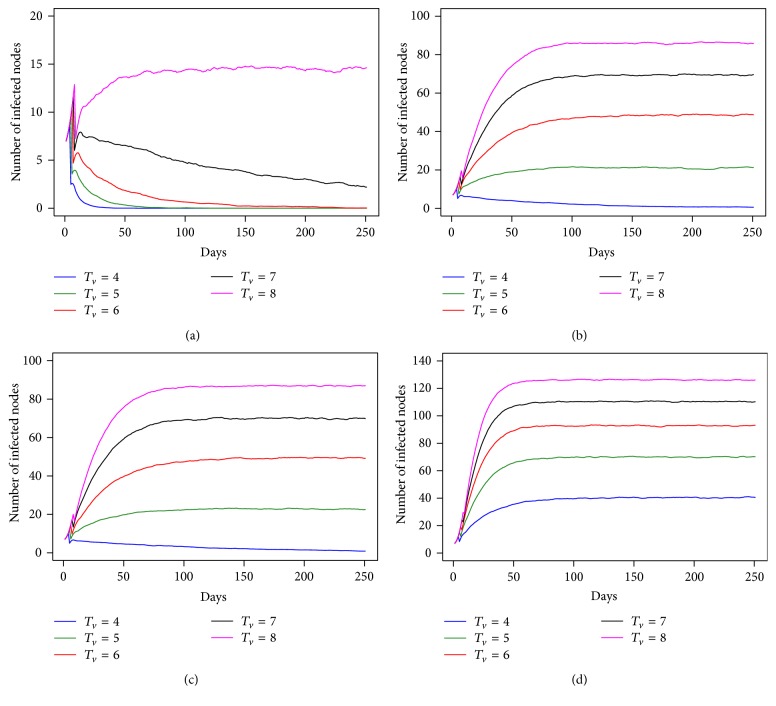
The number of infected nodes as a function of time for *T*_*v*_ = 4 to 8 days under difference transmission probability ((a) *P*_HT_ = 0.3 and *P*_HH_ = 0.3, (b) *P*_HT_ = 0.5 and *P*_HH_ = 0.3, (c) *P*_HT_ = 0.5 and *P*_HH_ = 0.5, and (d) *P*_HT_ = 0.7 and *P*_HH_ = 0.5) when the first infected node placed is at villages.

We found that the dynamics of the disease were specified by two parameters, the transmission probability and the virus inactivation period.  Figure 2 shows the result of individual-based model for the spread of avian influenza without intervention strategies when the first infection takes place at villages (1.00% of the villages or four infected villages). At the low transmission probability of both *P*_HT_ and *P*_HH_, we found that *T*_*v*_ did not affect the spread of the disease. The number of infected nodes decreased over the timeframe *T*_*v*_ = 4–7 days. This suggests that an epidemic would not occur. However, *T*_*v*_ had an impact on the epidemic at *T*_*v*_ = 8 days. In Figures 2(b) and 2(c) at *T*_*v*_ = 4 days, the number of infected nodes started to decrease with a similar pattern as shown in Figure 2(a), although the transmission probability is higher. However, at *T*_*v*_ ≥ 5, the number of infected nodes increased. It is therefore clear that *T*_*v*_ affects the size of the epidemic and also the number of infected nodes. At high *T*_*v*_, the virus can persist longer in the environment and increase the probability of infection, leading to the spread of the disease. With high transmission probability, *T*_*v*_ affects the size of the epidemic, with the number of infected nodes increasing and approaching steady state as in Figure 2(d).

**Figure 3:**
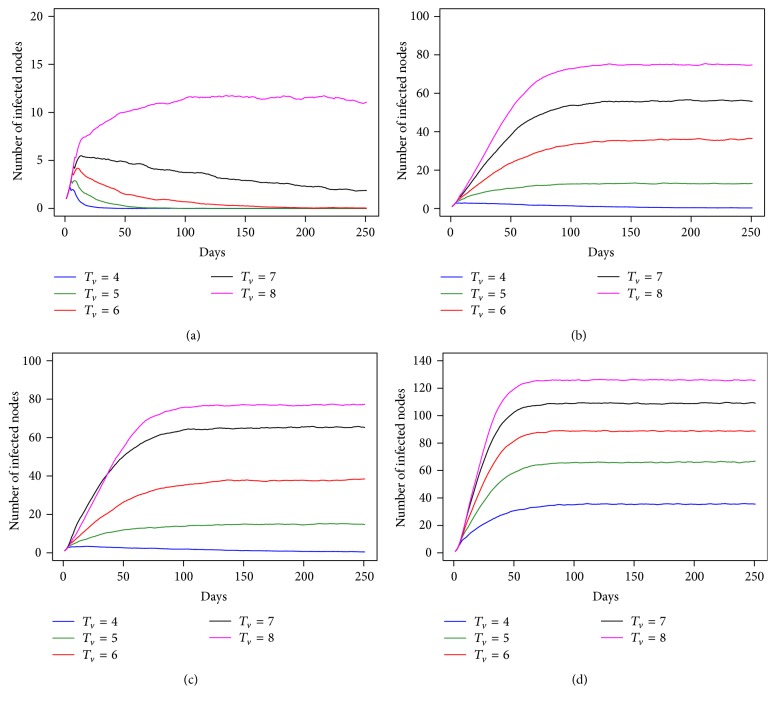
The number of infected nodes as a function of time for *T*_*v*_ = 4 to 8 days under difference transmission probability ((a) *P*_HT_ = 0.3 and *P*_HH_ = 0.3, (b) *P*_HT_ = 0.5 and *P*_HH_ = 0.3, (c) *P*_HT_ = 0.5 and *P*_HH_ = 0.5, and (d) *P*_HT_ = 0.7 and *P*_HH_ = 0.5) when the first infected node is a trader of trader.

In this study, we also initialized the first infection at an individual trader of trader with the amount equal to the proportion of the first infection at villages. The result from the individual-based model is shown in Figure 3. The dynamic behavior of the epidemic shows a similar pattern as in Figure 2, but with a different number of infected nodes and a different length of time to reach the maximum number of infected nodes. The period of time required for the number of infected nodes to increase to the maximum is longer than that indicated in the results from the first infection at villages under the same transmission probability. To compare clearly, we compared the straight line or the slope before approaching the steady state in Figure 2(b) with Figure 3(b) at *T*_*v*_ = 6–8 days; at this *T*_*v*_ it is implied that an epidemic was in fact occurring. We found that the number of infected nodes finally tends to reach a steady state at 65 days with *T*_*v*_ = 6, 50 days and with *T*_*v*_ = 7, and 46 days at *T*_*v*_ = 8, as shown in Figure 2(b), while in Figure 3(b) the number of infected nodes finally tends to steady state at 93 days with *T*_*v*_ = 6, 84 days and with *T*_*v*_ = 7 and 72 days at *T*_*v*_ = 8. This result indicates that the speed of the spread of the disease illustrated in Figure 2 is faster than that in Figure 3. The difference of the first infection between villages and trader of traders has an effect according to the period of time that it took to reach the steady state.

A sensitivity analysis was conducted for infection spread which considers the transmission probabilities *P*_HT_ and *P*_HH_. The actual transmission rate of avian influenza in the traditional trade network was not known; thus, the transmission probability was varied for this individual-based model. The result shows that the HPAI H5N1 spreads rapidly when *P*_HT_ increased. We also found that when *P*_HT_ increased, not only number of infected nodes at each time step rose up, but also the total number of infected nodes rose. The results indicated that we can classify the transmission probability for this individual-based model into 3 types: (1) low transmission probability, (2) moderate transmission probability, and (3) high transmission probability. For transmission probability *P*_HH_, we found that when *P*_HH_ increased, the number of infected nodes increased. The outcomes shown in Figure 2(b) are close to those illustrated in Figure 2(c). This suggests that *P*_HH_ impacts the dynamics of epidemics less than *P*_HT_. The result for Figure 3 is similar to the result for Figure 2; *P*_HH_ still has less impact on the epidemics.

**Figure 4:**
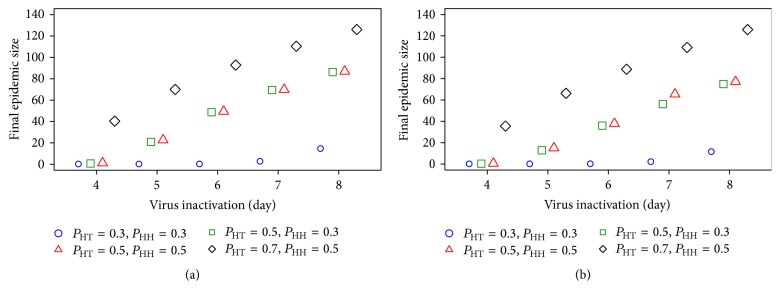
The final epidemic size as a function of *T*_*v*_ when the first infection is at villages (a) and a trader of trader (b).

We subsequently considered the effect of virus inactivation.  Figure 4 shows the impact of virus inactivation on the final epidemic size. The final epidemic size is defined as the number of infected nodes at steady state [34]. We found that *T*_*v*_ increased as the final epidemic size increased, so the final epidemic size is a function of *T*_*v*_. In Figure 4 (both upper and lower), the final epidemic sizes rapidly increased at high transmission probability whereas the results of low transmission probabilities, *P*_HT_ = 0.3 and *P*_HH_ = 0.3, were only slightly changed. These results confirm that *T*_*v*_ is less important for the spread of HPAI H5N1 at low transmission probability while, at high transmission probability, *T*_*v*_ is more important. The result of Figure 4 also confirms the results of Figures 2 and 3. When *P*_HT_ increases, the number of infected nodes and the final epidemic size rapidly increase. In contrast, when *P*_HH_ increases the number of infected nodes and the final epidemic size remains unchanged. Thus, *P*_HT_ is more important for the speed of spreading and final epidemic size than *P*_HH_.

### 3.2. Avian Influenza Spread with Intervention Strategies

**Figure 5:**
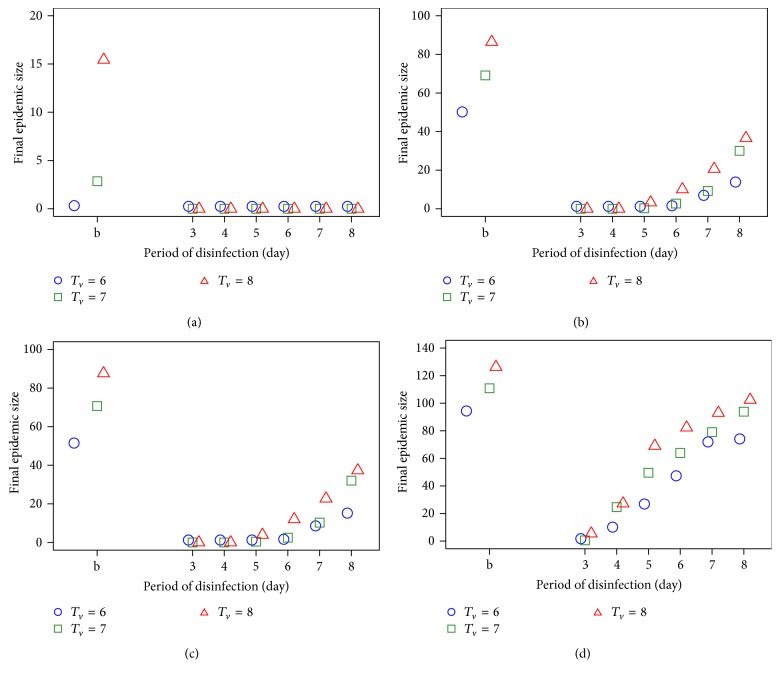
The final epidemic size as a period of disinfection for *T*_*v*_ = 6 to 8 days under difference transmission probability ((a) *P*_HT_ = 0.3 and *P*_HH_ = 0.3, (b) *P*_HT_ = 0.5 and *P*_HH_ = 0.3, (c) *P*_HT_ = 0.5 and *P*_HH_ = 0.5, and (d) *P*_HT_ = 0.7 and *P*_HH_ = 0.5) for the first infection at villages. b is the baseline of no disinfection period.

**Figure 6:**
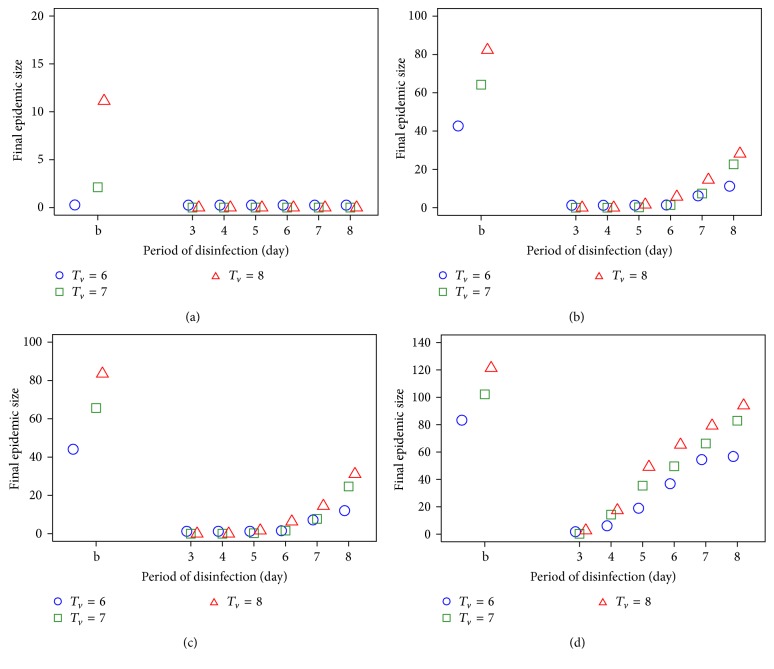
The final epidemic size as a period of disinfection for *T*_*v*_ = 6 to 8 days under difference transmission probability ((a) *P*_HT_ = 0.3 and *P*_HH_ = 0.3, (b) *P*_HT_ = 0.5 and *P*_HH_ = 0.3, (c) *P*_HT_ = 0.5 and *P*_HH_ = 0.5, and (d) *P*_HT_ = 0.7 and *P*_HH_ = 0.5) for the first infection at a trader of trader. b is the baseline of no disinfection period.

To develop effective strategies for preventing virus transmission and controlling the spread of the virus, we have also performed intervention strategies on the traditional trade networks. For intervention strategies, we chose *T*_*v*_∈ (6 d, 7 d, 8 d) because the spread of disease still occurred at high *T*_*v*_ so the effect of *T*_*c*_ can be explicit. To reduce the disease spreading through the traditional trade network, virus disinfection could be applied simultaneously to household traders and trader of traders. This intervention was shown to reduce the final epidemic size when compared with baseline or no disinfection period (Figures 5 and 6). The relationship between the final epidemic size and the period of virus disinfection, when the first infection occurs at villages, is shown in Figure 5. We found that the impact of virus disinfection on the final epidemic size is less important for lower transmission probability (Figure 5(a)) with fewer infected nodes. For medium transmission probability (Figures 5(b) and 5(c)), the impact of virus disinfection on the final epidemic size was reduced. At a high level of transmission probability, the final epidemic size increased as *T*_*c*_ increased. The most efficient disinfection intervention for this high level of transmission probability is at *T*_*c*_ = 3 days.

For the first infection at a trader of trader, we found that the virus disinfection has less impact on the final epidemic size at a low transmission probability as shown in Figure 6. The disease dynamics are also similar to the dynamics for the villages, as discussed above. We found that the disinfection process should be applied frequently and thoroughly to have a substantial impact on disease spread. If disinfection is sequentially applied less frequently and less thoroughly, the benefit of this intervention is lost. One of the important factors that impact the final epidemic size is the transmission probability. In both Figures 5 and 6, when transmission probability increases, the final epidemic sizes are high especially with the increasing of transmission probability *P*_HT_. The transmission probability has an impact not only on the final epidemic size but also on the period of virus disinfection. At low transmission probability, the period of virus disinfection has less effect on final epidemic size because at low probability, it cannot cause the disease spread and epidemics therefore do not occur. In high transmission probability situations, the period of virus disinfection has effect on the final epidemic size, which decreased in a short period of time.

**Figure 7:**
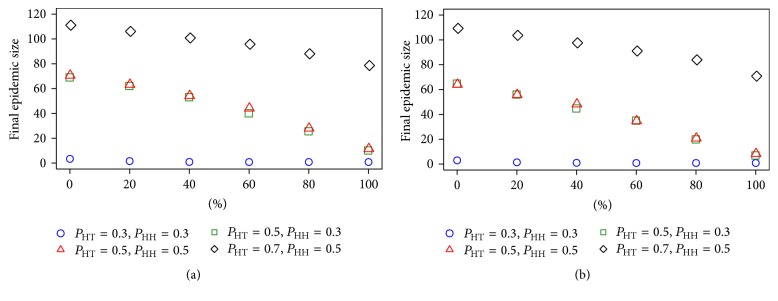
The final epidemic size as a proportion of disinfected household trader and trader of trader when the first infection occurred at villages (a) and a trader of trader (b) under difference transmission probability.

The secondary factor in the spread of avian influenza is mainly achieved through human-related activities such as the movement of staff, vehicles, and equipment [3]. To reduce the disease spread through the traditional trade network, disinfection may apply to the traders' vehicles and equipment but, in reality, we cannot disinfect 100% of the virus. In this study, we also considered the amount of disinfected nodes as a percentage of disinfection. The results of various percentages of disinfection are shown in Figure 7. We found that the final epidemic size also reduced as a result of the increasing percentages of disinfection. The percentage of virus disinfection found to be effective for every transmission probability between 80% and 100%. However, such percentages of virus disinfection are not significantly different at moderate transmission probability. At low transmission probability (*P*_HT_ = 0.3, *P*_HH_ = 0.3), the percentages of virus disinfection hardly impact the final epidemic size because the spread of the disease does not occur.

The number of infected nodes with and without an intervention strategy under transmission probabilities *P*_HT_ = 0.5 and *P*_HH_ = 0.5 are compared in Table 1. We also placed the first infected nodes and disinfected nodes at different types of member in the network. We found that whenever the epidemics occurred, the disinfection at both HT and TT is the best solution. This strategy can disinfect the virus at the first infected nodes V, HT, TT, and HT and TT by 85, 88, 87, and 87 percent, respectively. The results in this study reasonably answer the question why we must clean both HT and TT. This result also confirms our hypothesis that cleaning a hub in the network is a reasonable strategy against viruses as the hubs spread the virus faster than any other nodes.

## 4. Discussion

The traditional trade network in Phitsanulok, Thailand, appeared to be well connected based on the activities of traders buying chickens from villages and supplying urban markets with chicken meat [24]. To better understand the dynamics of avian influenza in the traditional trade network, an individual-based avian influenza model was constructed on this network. The influence of virus inactivation and the impact of transmission probability on epidemic dynamics were studied. Our results suggest that virus inactivation impacted the final epidemic size. This finding is similar to that of previous research in Northern Vietnamese Live Bird Markets (LBMs) [26]. In addition to the long period of virus inactivation, the transmission probability is one of the important factors. In this study, we classify the transmission probability into 3 types: (1) low transmission probability, (2) moderate transmission probability, and (3) high transmission probability.

*P*_HT_ is the transmission probability that household traders may become contaminated through contact with contaminated villages or trader of traders. This indicates that their relationship encourages a faster spread of the disease [9]. In other words, the transmission probability *P*_HH_, which is the transmission probability that a household trader becomes infected through contact with contaminated household traders when visiting the same trader of trader or the same village, has less impact on epidemics. The household traders have less chance to contact other household traders at the same place in the same time, as there is no reason for traders to sell chickens to other traders. It is therefore possible that the spread of avian influenza between household traders may play a less significant role.

Two scenarios of the first infected nodes were studied including villages and a trader of trader with the same proportion. Trader of traders have a high clustering and degree correlations, while, in the villages, they raise chickens that do not have contact with one another other than through the business of the traders [24], since villages have a low degree of the node. However, this kind of farming system is at high risk for the spread of disease [9]. The results show that when the first infection occurred at either a village or a trader of trader, the dynamic behavior of the epidemic follows a similar pattern. However, when there are different numbers of infected nodes and different length of time for the maximum number of nodes to become infected, the pattern of the epidemic may change. In Figures 2 and 3, with reference to trader of traders, 1.00% refers to one infected trader of trader. With regard to villages, 1.00% refers to four infected villages. This indicates that trader of traders play an important role in the spread of avian influenza. In fact, live bird market in Thailand is not common, and most chickens are killed before being sent to the market place. Trader of traders in this case may act as a major source of the virus because these traders gather live chickens together before slaughter and sell them to the markets. The practices of trader of traders are therefore similar to what happens in live bird markets in other Asian counties like Cambodia [35], China [36], and Vietnam [37].

The spread of avian influenza virus in Thailand has serious consequences, particularly for poultry production and the livelihood of farmers [10]. During this project, we developed a number of intervention strategies to prevent the uncontrolled spread of the disease. As the household trader and trader of trader spread the virus faster than any other nodes, the cleaning hub strategy (household trader and trader of trader) is a reasonable strategy against the propagation of the virus [32]. This strategy, not only reduced the number of infected individuals, but also decelerated the spread of the disease. This suggests that the virus spreads less rapidly in networks where the hubs had been disinfected, as against networks where the hubs had not been disinfected (see Table 1). Apparently, contaminated traders act as hubs, spreading the virus through the traditional trade network. Nonetheless, the effectiveness of the strategy of disinfecting hubs depends on the survival of the virus in the environment. A stronger or more vigorous virus may be more difficult to destroy through a disinfection campaign, and the virus may survive in the environment [32]. From an economic perspective, the cleaning hub strategy is cost effective, as it costs less than attempting to clean chicken runs in the villages [38]. The disinfection strategy may be easy to implement as it is in keeping with the existing public health measures. For these measures to be effective, public education may be necessary to convince and support the disinfectant of trader people as a disinfection strategy. Trader people may also need to change their behavior in order to help prevent the spread of diseases. This may prove to be impractical, at least in the shorter term, as people may prove to be unwilling to change their behavioral patterns that have existed for generations. To make this intervention sustainable is, indeed, very challenging.

In conclusion, we would like to suggest that the individual-based model may play an important role in comprehending how diseases spread in this kind of network and how to halt the chain of transmission. By using the computational results, we are able to understand the effect of transmission probability and virus inactivation and also investigate the appropriate intervention strategy. The results suggest that cleaning the equipment of household traders and disinfecting trader of traders leads to a decrease in the transmission of diseases and epidemic size. To prevent and control the spread of avian influenza among backyard chicken populations, intervention strategies applied to the pathways can be beneficially promoted, in order to control the spreading of avian influenza in these networks.

However, it is acknowledged that this model has several limitations. The model did not consider distance between nodes in the network and other natural factors such as the aquatic biotopes (water, mud) [33], which influence the survivability of avian influenza virus in the environment. The roles of migration of wild birds or other domestic bird movements on the network also need to be further investigated [5]. The behavior of chicken movement across villages may increase the chance of epidemic and therefore it makes the disinfection strategy less effective. In this model, the traders use vehicles (motorcycles and pick-up trucks) in the movement and contact. These vehicles enable them to move rapidly between villages and trader of traders. The distance between nodes may not be very important, especially if the distances are short. The data sources are only from a single source, which may be insufficient [24]. This bias may be introduced into the traditional trade network in Phitsanulok Province, in which the household trader is the main or the only mediator in contact among villages and trader of trader. However, in situations where the available data is sufficient, this model should be proven to be useful and also be applicable to traditional trade networks in other provinces.

Although HPAI H5N1 has not been reported in human in Thailand since 2007 and the last outbreak of HPAI H5N1 in this country was notified in 2008, the virus still circulates in poultry populations in Thai neighboring countries including Cambodia, Myanmar, and Vietnam [39]. The number of confirmed human cases was reported in Indonesia until now [40]. In 2015, the number of confirmed human cases worldwide increased leading to the concern of the reemergence of this disease [40]. Therefore, the risk of HPAI H5N1 reintroduction in Thailand has not yet been eliminated. Our proposed model can be used to describe the dynamics of avian influenza on the traditional trade networks in Phitsanulok Province, Thailand. This model also can be applied to other traditional trade networks in other provinces, where the network is a scale-free network. The intervention strategies in our proposed model can be used for the H5N1 reemergence or a new atypical influenza virus subtype H7N9, which may be introduced in Thailand. Moreover, the model can also be applied to other emerging diseases in the poultry in the future.

## Figures and Tables

**Table 1:** The percentage of infected nodes reduction compared with no virus disinfection process.

Disinfected node	First infected node			
	V	HT	TT	HT & TT
V	75.44%	84.97%	77.93%	84.08%
HT	69.01%	73.00%	74.38%	71.08%
TT	3.51%	6.96%	5.17%	3.32%
HT and TT	84.87%	88.22%	87.31%	86.76%
